# A New Preparation of Gutta-Percha

**Published:** 1892-04

**Authors:** William H. Rollins

**Affiliations:** Boston


### A NEW PREPARATION OF GUTTA-PERCHA.

BY WILLIAM H. ROLLINS, BOSTON.

In setting crowns of porcelain with platinum pins extending into the roots, and for setting gold crowns and caps, I find a filling made of vermilion and gutta-percha of service.

This is readily made by mixing together with heat and careful working one part of gutta-percha and three parts of vermilion. This combination resists the destructive action of the mouth much better than the usual combination of gutta-percha and oxide of zinc. For buccal cavities, where the ordinary gutta-percha filling softens on the surface, it is of value. A whole list of gutta-percha stoppings can be prepared without the use of oxide of zinc, which are interesting from an experimental point of view, and I shall hope at some future time to report on these. The combination of iron oxide with gutta-percha is one of these, and favorable results seem to have been obtained with this mixture, but it is a matter of years to determine the relative value of these fillings.

